# Matrix metalloproteinase-2 is downregulated in sciatic nerve by streptozotocin induced diabetes and/or treatment with minocycline: Implications for nerve regeneration

**DOI:** 10.1016/j.expneurol.2014.08.017

**Published:** 2014-11

**Authors:** Sumia Ali, Heather E. Driscoll, Victoria L. Newton, Natalie J. Gardiner

**Affiliations:** Faculty of Life Sciences, University of Manchester, Oxford Road, Manchester M13 9PT, UK

**Keywords:** Neuropathy, Dorsal root ganglia, Extracellular matrix, IENF, STZ, NGF, DRG, ECM

## Abstract

Minocycline is an inhibitor of matrix metalloproteinases (MMPs) and has been shown to have analgesic effects. Whilst increased expression of MMPs is associated with neuropathic pain, MMPs also play crucial roles in Wallerian degeneration and nerve regeneration. In this study we examined the expression of MMP-2, MMP-9 and tissue inhibitor of metalloproteinase (TIMP)-1/-2 in the sciatic nerve of control and streptozotocin-induced diabetic rats treated with either vehicle or minocycline by quantitative PCR and gelatin zymography. We assessed the effects of minocycline on nerve conduction velocity and intraepidermal nerve fibre (IENF) deficits in diabetic neuropathy and investigated the effects of minocycline or MMP-2 on neurite outgrowth from primary cultures of dissociated adult rat sensory neurons.

We show that MMP-2 is expressed constitutively in the sciatic nerve *in vivo* and treatment with minocycline or diabetes leads to downregulation of MMP-2 expression and activity. The functional consequence of this is IENF deficits in minocycline-treated nondiabetic rats and an unsupportive microenvironment for regeneration in diabetes. Minocycline reduces levels of MMP-2 mRNA and nerve growth factor-induced neurite outgrowth. Furthermore, *in vivo* minocycline treatment reduces preconditioning-induced *in vitro* neurite outgrowth following a sciatic nerve crush. In contrast, the addition of active MMP-2 facilitates neurite outgrowth in the absence of neurotrophic support and pre-treatment of diabetic sciatic nerve substrata with active MMP-2 promotes a permissive environment for neurite outgrowth. In conclusion we suggest that MMP-2 downregulation may contribute to the regenerative deficits in diabetes. Minocycline treatment also downregulates MMP-2 activity and is associated with inhibitory effects on sensory neurons. Thus, caution should be exhibited with its use as the balance between beneficial and detrimental outcomes may be critical in assessing the benefits of using minocycline to treat diabetic neuropathy.

## Highlights

• MMP-2, but not MMP-9, is constitutively expressed in the adult rat sciatic nerve.
• Levels of cleaved active MMP-2 are reduced in sciatic nerve of diabetic rats.
• Active MMP-2 potentiates neurite outgrowth from sensory neurons.
• Minocycline reduces levels of MMP-2 mRNA and impairs NGF-induced neurite growth.
• Minocycline did not prevent nerve dysfunction in experimental diabetic neuropathy.

## Introduction

Diabetic neuropathy (DPN) is a common secondary complication of diabetes mellitus, which is associated with biochemical and structural changes in the nervous system including nerve conduction velocity (NCV) deficits and altered mechanical and thermal sensitivity (Tomlinson and Gardiner, 2008). A common feature of both clinical and experimental diabetic neuropathy is degeneration of distal nerve fibres and a reduced capacity for regeneration of injured axons (Beiswenger et al., 2008, Christianson et al., 2006, Kennedy and Zochodne, 2000, Kennedy and Zochodne, 2005). This leads to numbness, loss of protective sensation and an increased risk of amputation. Other than glycaemic control there is no effective treatment and current strategies which treat neuropathic pain do not encourage neuronal repair (Tesfaye et al., 2013). There is therefore an unmet clinical need for effective treatments for DPN targeting both nerve regeneration and neuropathic pain.

Minocycline is a semi-synthetic analogue of tetracycline and a widely prescribed generic drug used to treat microbial infections. It has been shown to have anti-inflammatory, neuroprotective, anti-oxidative, and/or analgesic effects in a number of disease models and this paired with the good safety record of minocycline in patients has paved the way for clinical studies in a number of neurodegenerative diseases (Plane et al., 2010). The mechanisms underlying the neuroprotective effects of minocycline are unclear since minocycline inhibits multiple targets including matrix metalloproteinase (MMP)-2 and MMP-9. The upregulation of MMPs has been linked to the development and maintenance of neuropathic pain (Kawasaki et al., 2008). In experimental diabetic neuropathy the analgesic potential of minocycline has been explored (Bhatt and Veeranjaneyulu, 2010, Morgado et al., 2011), but none of these studies have investigated the effects on regeneration.

MMPs are a large family of zinc-dependent proteolytic enzymes, best known for their role in tissue remodelling, degradation of extracellular matrix (ECM) molecules, release of ECM-sequestered growth factors and cleavage and activation of membrane-bound cytokines and their receptors (Sternlicht and Werb, 2001). Expression and activity of MMPs are tightly regulated at three levels: by transcriptional regulation; by activation — they are secreted in an enzymatically inactive state as pro-enzymes which are activated in a proteolytic manner by direct cleavage of the pro-peptide by another MMP or a non-proteolytic manner by organomercurials; and by inhibition *via* interactions with tissue inhibitors of MMPs (TIMPs) (Sternlicht and Werb, 2001).

In the peripheral nervous system the coordinated expression of ECM, MMPs and TIMPs during Wallerian degeneration and nerve regeneration governs the success of nerve repair (Gantus et al., 2006). Following nerve injury, there are spatial and temporal alterations in the expression of MMPs. MMP-9 is rapidly and transiently upregulated at sites of peripheral nerve injury (Demestre et al., 1999, Ferguson and Muir, 2000, Shubayev and Myers, 2000) with increased expression in Schwann cells, blood vessels, and activated macrophages. MMP-9 facilitates blood nerve barrier breakdown enabling macrophage and neutrophil infiltration (Siebert et al., 2001) and is essential to Wallerian degeneration (Hughes et al., 2002, Ide, 1996, Johnson et al., 2005, Mantuano et al., 2008). MMP-2 mRNA and protein levels increase later and remain upregulated up to 63 days post-nerve crush. Early MMP-2 expression is in Schwann cells and endoneurial cells (Demestre et al., 2004, La et al., 1996, Muir, 1994) whilst later MMP-2 is increased within the regenerating axons and degrades inhibitory ECM components promoting a permissive environment for regeneration (Ferguson and Muir, 2000, Zuo et al., 1998). MMP-2 knockout mice show reduced regeneration of axons following spinal contusion injury and impaired functional recovery (Hsu et al., 2006) highlighting the importance of MMPs for successful regeneration.

A number of studies have described an increase in systemic MMPs in diabetes (Jacqueminet et al., 2006) and altered expression of MMPs has been implicated in the pathophysiology of a number of secondary complications including nephropathy (Thrailkill et al., 2009) and poor wound healing (Liu et al., 2009). To date, there is little information about the expression or activity of MMPs and/or their role in the regenerative deficits associated with diabetic neuropathy. The aims of this study were (1) to compare the expression of MMP-2, MMP-9 and TIMP-1/2 in the peripheral nerve of control rats and rats with streptozotocin (STZ)-induced diabetes; (2) to assess the effects of minocycline on indices of experimental diabetic neuropathy and regulation of MMPs in the peripheral nerve; and (3) to investigate the effect of MMP-2 and minocycline on *in vitro* sensory neuron growth.

## Materials and methods

### Reagents

All chemicals were obtained from Sigma-Aldrich (Poole, Dorset, UK) unless otherwise stated.

### Animal studies

All animal studies and procedures were licensed under the UK Animals (Scientific Procedures) Act 1986. Diabetes was induced in adult male Wistar rats (250–325 g, Charles River, UK) with freshly dissolved STZ (55 mg/kg in sterile saline, i.p.), administered following an overnight fast. Diabetes was verified 3 days post-STZ (OptimumPlus; MediSense, UK) and rats with blood glucose > 15 mmol/l were classified as diabetic. STZ-diabetic and age-matched control rats were maintained for 4–12 weeks. For the minocycline-prevention study: rats were randomly allocated into one of four treatment groups (control-vehicle (*n* = 8); control-minocycline (*n* = 8); diabetic-vehicle (*n* = 10); diabetic-minocycline (*n* = 10)). Minocycline was administered by daily gavage rather than the intraperitoneal route to avoid deposition of minocycline in the peritoneal cavity, which has been associated with inflammatory lesions and variability in absorption (Fagan et al., 2004). Treatment with minocycline 25 mg/kg, p.o or vehicle (water, p.o.) commenced 3 days post-STZ, and continued daily for the duration of the trial. Animals were monitored daily, weighed twice weekly and maintained for 8 weeks post-STZ. Body weight and blood glucose data are shown in Table 1.

**Table 1:** Diabetic rats are significantly lighter and hyperglycaemic compared to age-matched nondiabetic control rats. Minocycline had no significant effect on body weight or terminal blood glucose levels of control or diabetic rats (note, 1/10 rats from the diabetic-vehicle group and 5/10 rats from the diabetic-minocycline group became normoglycaemic between 4 and 8 weeks post-STZ — their data was excluded from all analysis). For blood glucose levels greater than the upper limit of detection of glucose meter, data was assigned as 27.8 mmol/l. Data represents mean ± s.d. ***p < 0.001 *t*-test and two-way ANOVA followed by Bonferroni posthoc tests.

Duration (weeks)	Animal group (*n* numbers)	Start body weight (g)	End body weight (g)	End blood glucose (mmol/l)
*Timecourse study*				
4	Age-matched control (10)	340 ± 9	471 ± 7	8.1 ± 1
	Diabetic (12)	339 ± 7	370 ± 14^⁎⁎⁎^	27.8 ± 0^⁎⁎⁎^
8	Age-matched control (10)	335 ± 5	579 ± 18	9.1 ± 1
	Diabetic (12)	343 ± 3	383 ± 10^⁎⁎⁎^	27.8 ± 0^⁎⁎⁎^
12	Age-matched control (10)	334 ± 6	612 ± 14	9.9 ± 1
	Diabetic (12)	342 ± 5	396 ± 11^⁎⁎⁎^	27.8 ± 0.0^⁎⁎⁎^

*Minocycline study:*				
8	Nondiabetic-vehicle (8)	358 ± 20	580 ± 39	5.3 ± 0.8
	Nondiabetic-minocycline (7)	364 ± 20	567 ± 63	6.8 ± 2.3
	Diabetic-vehicle (9)	366 ± 19	383 ± 50^⁎⁎⁎^	27.7 ± 0.0^⁎⁎⁎^
	Diabetic-minocycline (5)	365 ± 12	431 ± 81^⁎⁎⁎^	27.5 ± 0.2^⁎⁎⁎^

An additional group of adult male Wistar rats (250–300 g) was daily dosed with either minocycline (25 mg/kg p.o, *n* = 4) or vehicle (water, p.o, *n* = 4) for 3 days. Rats were then anaesthetized with isoflurane (2% in oxygen) and, under sterile conditions, the left sciatic nerve was exposed at mid-thigh level. The sciatic nerve was crushed with the tips of watchmaker's forceps (2 × 15 s) the wound was closed in layers and the animals recovered under observation. Dosing continued daily for a further 3 days, then rats were killed and ipsilateral and contralateral lumbar (L)4/5 dorsal root ganglia (DRG) were removed for cell culture and sciatic nerves removed for PCR 3 h post last dose of minocycline.

### Nerve conduction velocity (NCV)

One day following the last dose of minocycline, rats were terminally anaesthetized with isoflurane (2–4% in oxygen) and electromyograms were recorded from plantar foot muscles on a Powerlab 4 with ABI Scope software in response to stimulation (1.5–5 V, 2 ms pulses, Powerlab 4) at the sciatic notch and Achilles tendon. The length of nerve between the two stimulus points was measured *ex vivo*. Motor NCV was calculated using the latencies of compound M waves, and sensory NCV was calculated from H reflex latencies.

### Tissue processing

Rats were killed by anaesthetic overdose and the L4/5 DRG, plantar surface of hindpaws and sciatic nerves were rapidly dissected and either snap frozen on dry ice, or post-fixed for 4 h in 4% paraformaldehyde (in 0.1 M phosphate buffer (PB)), cryoprotected (10–30% sucrose in 0.1 M PB at 4 °C over 48 h), frozen in OCT-embedding medium (VWR, UK) and then stored at − 80 °C.

### Sensory neuron culture

Dissociated sensory neurons were prepared from DRG obtained from either L4/5 DRG or all vertebral levels of adult male Wistar rats (200–250 g, Charles River, UK). Sensory neurons were prepared as previously described (Gardiner et al., 2005). Neurons were then either seeded onto repeatedly freeze-heated longitudinal sections of sciatic nerve (Ferguson and Muir, 2000) obtained from control or diabetic rats (8 week post-STZ) or onto laminin (2 μg/ml)-coated 8-well Lab-Tek chamber slides (VWR, UK; (Gardiner et al., 2005)) and then treated with combinations of: active MMP-2 (100–200 ng/ml); nerve growth factor (NGF; 10 ng/ml); and minocycline (1–50 μM) for 18–48 h. Media were removed for analysis using a commercial NGF ELISA kit (NGD E_max_ Immunoassay G7330, Promega UK). Sensory neurons were either lysed in ice-cold lysis buffer (0.5 M Tris, pH 7.6, 0.2 M NaCl, 10 mM CaCl_2_, 1% Triton-X-100), or fixed with 2% paraformaldehyde for 15 min at room temperature.

### Gelatin zymography

Sciatic nerves were desheathed, homogenized in ice-cold lysis buffer (0.5 M Tris, pH 7.6, 0.2 M NaCl, 10 mM CaCl_2_, 1% Triton-X-100), and centrifuged (9000 *g*, 30 min at 4 °C) and the supernatant was collected. 20 μg of protein from the supernatant was separated on SDS-polyacrylamide gels (containing 4.13 mg/ml gelatin under non-reducing, non-denaturing conditions). SDS was removed by incubating gels in wash buffer (50 mM Tris–HCl, pH 7.6, 5 mM CaCl_2_, 1 μM ZnCl_2_, 2.5% Triton X-100) to renature enzymes. Gels were incubated in reaction buffer (wash buffer without detergent) for 72 h at 37 °C to stimulate gelatinolysis, then stained with Coomassie Brilliant Blue R-250 (0.1% diluted in 40% methanol and 10% acetic acid) for 1 h, then destained (in 10% methanol and 1% acetic acid) until clear gelatinolytic bands appeared. Gels were dried using DryEase Min-gel drying system (Invitrogen, UK), scanned and semiquantitative densitometry was performed on raw digitized images using SigmaScan Pro5 software (SPSS, Chicago, IL). For visual clarity within figures, the images were converted to greyscale and inverted (Adobe Photoshop), so digested bands appear as black on a white background.

### Real-time PCR

Total RNA was extracted from sensory neuron cultures using an RNeasy kit (Qiagen) and from desheathed sciatic nerve of control and STZ-diabetic rats (4, 8 and 12 weeks post-STZ) using the RNeasy Lipid Tissue Mini Kit (Qiagen). Genomic DNA was removed using the DNA-free kit (Ambion). Equal amounts of RNA were reverse transcribed using MMLV reverse transcriptase (Applied Biosystems) and quantitative PCR was carried out using SYBR green (Applied Biosystems). Reactions were set up in 96 well plates and each sample was assayed in duplicate, with the inclusion of a no template control (no cDNA) for each primer set. Plates were cycled on an ABI 7300 real-time PCR machine: 55 °C for 5 min, followed by 95 °C for 10 min, and 40 cycles of 95 °C for 15 s and 60 °C for 1 min. Data were normalized to cyclophilin B (PPIB). All primer pairs (see Table 2) were optimised at a 60 °C annealing temperature using traditional PCR prior to use in a real-time PCR system.

**Table 2:** **Forward and reverse primer sequences**. Primers were designed using Primer 3.0 and Blast primer programmes, PPIB was used as a reference (house-keeping) gene.

Primer	Forward primer sequence (5′-3′)	Reverse primer sequence (5′-3′)
MMP2	ATGGCATTGCTCAGGATCCGT	AGCCTTCTCTTCCTGTGGGG
MMP9	CTGCGTATTTCCATTCATCCTTCG	CGAGTTGCCCCCAGTTACAG
Timp1	TGGGGTGTGCACAGTGTTTC	CGCTCTGGTAGCCCTTCTCA
Timp2	CGGAAGGAGATGGCAAGATG	CCATCCAGAGGCACTCATCC
Il-6	ATATGTTCTCAGGGAGTCTTG	GTGCATCATCGCTGTTCATACA
TNF-α	GCCACCACGCTCTTCTGT	GGCAGCCTTGTCCCTTGA
PPIB	GGAGATGGCAGGAGGA	ACCACATCCATGCCTTCC

### Immunohistochemistry and immunocytochemistry

Tissue sections were washed in PBS, and non-specific binding was inhibited by incubation for 1 h in 5% normal donkey serum (in PBS containing 0.2% Triton-X-100 (P-BST)). Transverse sciatic nerve sections (12 μm) were immunostained with a combination of anti-MMP-2 (1:2000; Abcam, UK) and anti-S100β (1:500; Sigma, UK) overnight at 4 °C. Slides were washed in PBS-T and incubated with secondary antibodies (fluorescein isothiocyanate (FITC)-conjugated donkey anti-mouse and cyanine-3 (Cy-3)-conjugated donkey anti-rabbit (1:500)) for 1 h at room temperature. Transverse sections of hindpaw skin (20 μm) were immunostained overnight at 4 °C with anti-PGP9.5 (1:4000; AbD Serotec). Slides were washed and incubated with secondary antibody (donkey anti-rabbit Cy3; 1:1000) for 1 h at room temperature. Cultured sensory neurons were immunostained overnight at 4 °C with anti-β (III)-tubulin (1:500; Sigma) and with then donkey anti-mouse FITC (1:200). Following incubation in secondary antibodies (all from Jackson ImmunoResearch), slides were washed and mounted in Vectashield containing DAPI (Vector Labs, UK). Immunofluorescence was visualised using a Leica DMR fluorescence microscope, Hamamatsu camera and software or a Leica TCS SP5 AOB5 inverted confocal microscope using 100×/0.50 plan Flouotar objective and LASAF software.

### Image analysis

For analysis of neurite outgrowth, 20 randomly selected fields of view per well were used in order to calculate the percentage of neurite bearing cells (defined as neurons with neurites longer than 1.5 times the associated cell body diameter). Then, images of every neurite-bearing neuron in each Lab-Tek chamber, or on each individual sciatic nerve cryosection were acquired to calculate the length of the longest neurite and neurite density, using concentric ring analysis as previously described (Gardiner et al., 2005) using SigmaScan software (SPSS, UK). Data represent means ± standard deviation from independent culture experiments.

For analysis of intraepidermal nerve fibre density, the number of PGP-9.5-immunoreactive nerve fibre profiles which crossed the dermis/epidermis border was counted from 5 randomly selected fields of view from at least 5 sections per animal. Fibres that branched within the epidermis were counted as a single IENF. The length of the epidermis was measured using SigmaScan software (SPSS, UK) and data are expressed as mean density of IENF per unit length of epidermis (IENF/mm) ± standard deviation (Lauria et al., 2005).

Animals, tissues and cells were all analysed in a blinded fashion to prevent observer bias. Statistical analysis was conducted using GraphPad Prism 4.0 software using t-tests or one- or two-way ANOVA followed by Bonferroni's Multiple Comparison Tests as appropriate for the data set.

## Results

### Active MMP-2 is constitutively present in the sciatic nerve and is downregulated in diabetes

**Fig. 1:**
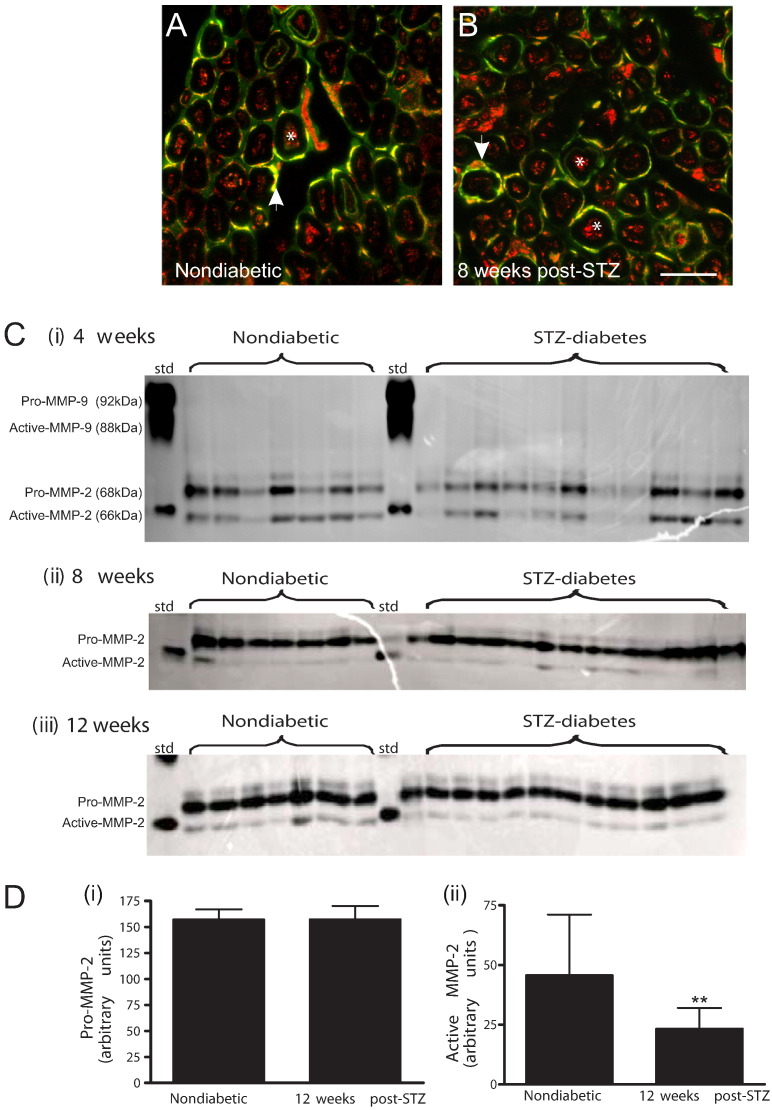
MMP-2 is expressed in axons and Schwann cells of the sciatic nerve of control and STZ-diabetic rats, but levels of active MMP-2 decrease in diabetes. MMP-2-immunoreactivity (red) was visualised in axons (asterisk) and Schwann cells (co-labelled with S-100β in green; arrows) of transverse sciatic nerve sections from (A) control and (B) STZ-diabetic rats. (C, i –iii) Proteolytic activity of MMP-2/-9 was assayed using gelatin zymography. Bands corresponding to molecular weight to the pro- and active-cleaved forms of MMP-2, were detected in sciatic nerve samples from control (*n =* 7 per timepoint) and diabetic animals (*n* = 11 per timepoint). In contrast, neither pro- nor active-MMP-9 were detected at any timepoint of diabetes. (D i) Densitometric analysis revealed no significant changes in levels of pro-MMP-2, however levels of active MMP-2 were significantly reduced at 12 weeks post-STZ (C iii, D ii; **p < 0.01, *t*-test). Each band represents sciatic nerve protein from an individual animal. Data represents mean + s.d. std = MMP-9 and MMP-2 protein standard to act as positive control/marker.

MMP-2-immunoreactivity was detected in both axons (asterisk, Fig. 1A) and Schwann cells (arrows, Fig. 1A) in the sciatic nerve of control rats, with no detectable change in cellular localisation at 4, 8 or 12 weeks of diabetes (Fig. 1B shows 8 weeks post-STZ).

Gelatin zymography is a sensitive method used to measure levels of both the latent proenzyme and the cleaved active enzyme (Platt et al., 2003). Protease activity in sciatic nerve samples from both age-matched control and diabetic rats was observed as digested bands in zymography gels, and corresponded in molecular weight to the latent proenzyme-form (68 kDa) and cleaved active-form (66 kDa) of MMP-2 (Fig. 1C). Neither pro-MMP-9 nor active MMP-9 was detected in sciatic nerve samples from diabetic or age-matched nondiabetic rats at any time point studied (Fig. 1C). There was no change in the levels of pro or active MMP-2 at 4 weeks post STZ. At 8 weeks post-STZ there was a trend towards a decrease in the amount of active MMP-2, this decrease was significant by 12 weeks post-STZ (p < 0.01, Fig. 1D). These data suggest that cleavage of the zymogen to the active enzyme may be compromised from 8–12 weeks post STZ.

### Active MMP-2 potentiates neurite elongation from sensory neurons

**Fig. 2:**
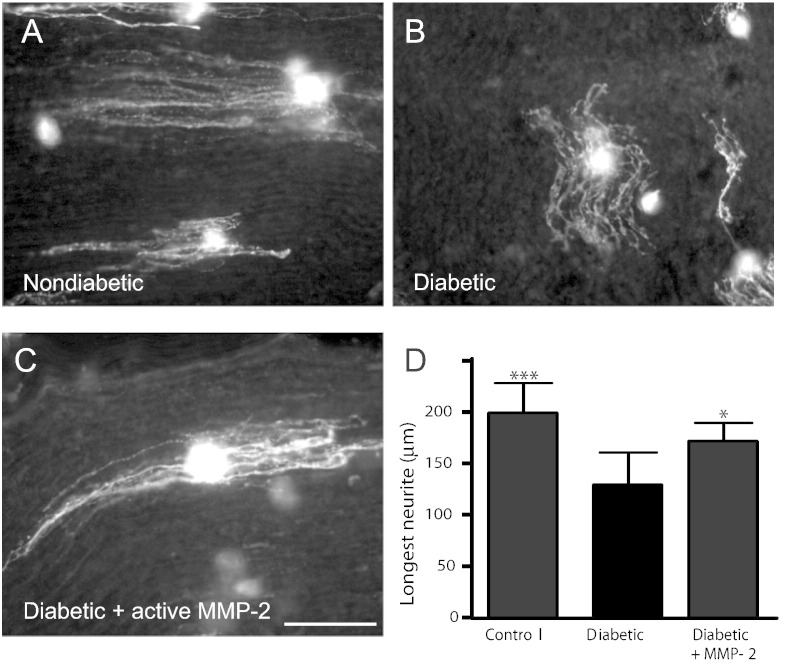
Neurite outgrowth on sciatic nerve cryosections from diabetic rats is increased following pre-treatment with active MMP-2. Sciatic nerve cryosections from control or diabetic rats (8 weeks post-STZ) were pretreated with vehicle or active MMP-2 (10 ng/ml; 3 h) and seeded with dissociated adult rat sensory neurons (NGF 10 ng/ml; 48 h). Neurons were visualised with GAP43. Neurite outgrowth on sciatic nerve cryosections from diabetic rats (B) was retarded compared to growth on control nerve (A), but was rescued by pretreatment with active MMP-2 (C, D; Data represents mean + s.d (*n* = 9–10 sections from *n* = 5 rats per condition)*;* *p < 0.05; ***p < 0.005 compared to diabetic nerve, one-way ANOVA followed by Bonferroni's Multiple Comparison Tests). Scale bar = 100 μm.

The functional consequence of this downregulation of active MMP-2 was investigated by seeding dissociated sensory neurons onto longitudinal sections of sciatic nerve obtained from control or STZ-diabetic rats (Fig. 2). Neurons plated onto control nerve substrata extended long well-organised neurites (Fig. 2A), whilst those plated on sciatic nerve substrata from diabetic rats extended shorter neurites that were less closely-associated with the Schwann cell basement membrane (Fig. 2B). Pre-treatment of nerve sections from diabetic rats with active MMP-2 (10 μg/ml; 3 h) significantly improved neurite extension (control: 199.5 ± 28 μm; diabetic: 129 ± 31 μm; diabetic pretreated with MMP-2: 180 ± 28 μm, *n* = 9–10 nerve sections from *n* = 5 rats per condition).

**Fig. 3:**
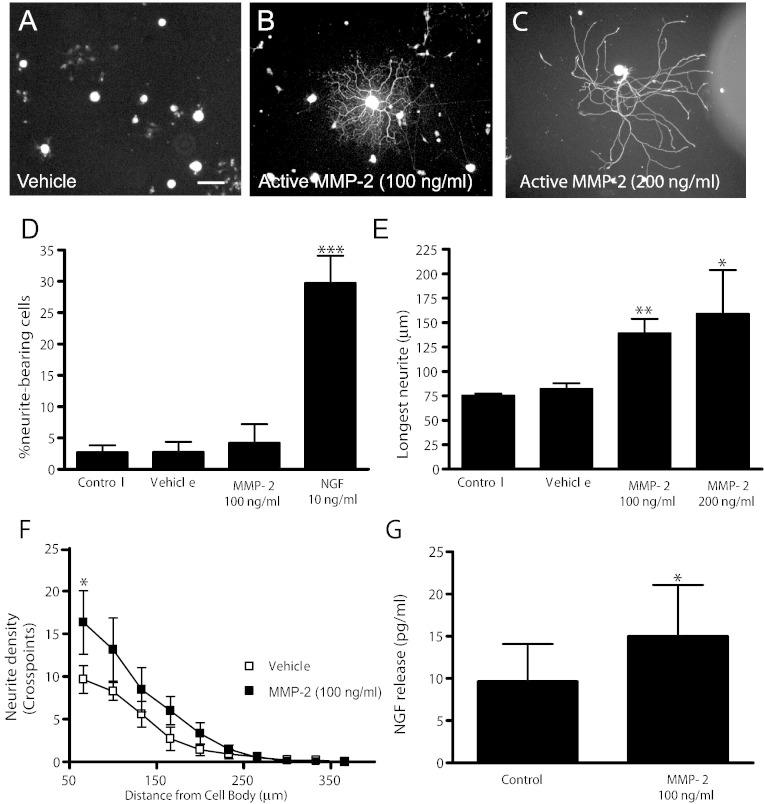
Active MMP-2 potentiates neurite outgrowth from adult rat sensory neurons. In the absence of neurotrophic support dissociated sensory neurons remained largely quiescent (A, D). Addition of active MMP-2 potentiated neurite outgrowth (B, C). The addition of active MMP-2 did not enhance (D) neurite initiation but significantly (E) increased the length of the longest neurite and (F) neurite density. (G) Active MMP-2 (100 ng/ml) increased the release of endogenous NGF. Data represent group mean ± s.d. from 4 independent cultures (*p < 0.05; **p < 0.01; ***p < 0.005) one-way (D–E) or two-way (F) ANOVA followed by Bonferroni's Multiple Comparison Tests or *t*-test compared to control (G). Scale bar = 100 μm.

In the absence of neurotrophic support, dissociated adult rat sensory neurons remained largely quiescent (Figs. 3A, D). The addition of active MMP-2 to neurons did not increase neuritogenesis (Fig. 3D) or alter cell survival (data not shown) but did potentiate neurite outgrowth of regenerating neurons (Figs. 3B–F). Neurons treated with MMP-2 extended significantly longer (control: 75 ± 2 μm *versus* active MMP-2 (100 ng/ml): 139 ± 15 μm, Fig. 3E, p < 0.01) and more dense neurites (Fig. 3F, p < 0.05). This facilitation of neurite outgrowth by the addition of active MMP-2 was associated with a significant increase in the levels of NGF in the media (Fig. 5G, p < 0.05).

These data suggest that active MMP-2 can enhance neurite outgrowth *via* remodelling of the ECM environment to one more favourable for regeneration, and by increasing the release of NGF.

### Minocycline treatment did not prevent large or small nerve fibre deficits in experimental diabetes

The analgesic efficacy of minocycline in diabetes is well characterised; therefore here we assessed the effects of minocycline on large and small fibres and on the regulation of MMPs in peripheral nerve in diabetic and age-matched control (nondiabetic) rats.

**Fig. 4:**
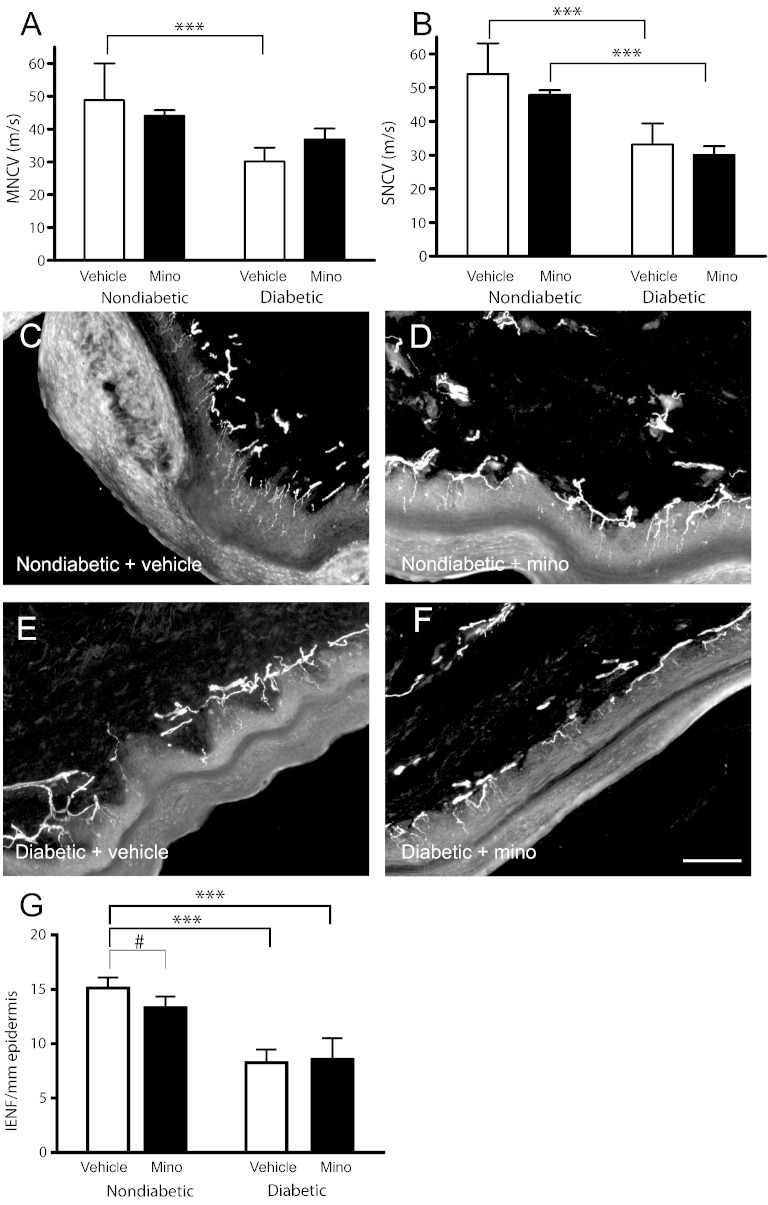
Effect of minocycline on indices of experimental diabetes. Rats were randomly assigned to treatment groups: age-matched control (nondiabetic) rats (vehicle (water p.o; *n* = 8) or minocycline-treated (25 mg/kg/day, p.o; *n* = 7)) and diabetic rats (vehicle (water, p.o; *n* = 9) or minocycline-treated (25 mg/kg/day, p.o; *n* = 5)). Rats were treated daily from 3 days post-STZ for 8 weeks. Sensory and motor NCV were measured under terminal anaesthesia at 8 weeks post-STZ (24 h post-minocycline). Minocycline had no significant effect on NCV in nondiabetic and diabetic rats (A, B). Data represent mean + s.d. (ns = not significant; *** p < 0.001; two-way ANOVA followed by Bonferroni's Multiple Comparison Tests). Vehicle-treated diabetic rats showed deficits in IENF density by 8-weeks post-STZ and minocycline-treatment did not protect against this loss (C–G). (D) Nondiabetic rats treated with minocycline showed IENF deficits compared to (C) vehicle-treated nondiabetics (G, ^#^p < 0.05 compared to vehicle-treated; ***p < 0.001 compared to nondiabetic rats; *n* = 5 per group; one-way ANOVA followed by Bonferroni's Multiple Comparison Tests).

Minocycline treatment did not cause significant changes in body weight or terminal blood glucose levels in diabetic or nondiabetic rats compared to vehicle-treated rats (Table 1). Vehicle-treated diabetic rats developed significant motor (Fig. 4A) and sensory (Fig. 4B) nerve conduction velocity (NCV) deficits compared to nondiabetic rats (p < 0.001). Treatment with minocycline did not significantly alter motor or sensory NCV.

There was a significant reduction in IENF in the plantar surface of the hindpaw of diabetic rats compared to nondiabetic rats (Figs. 4C, E, G; p < 0.001) and minocycline-treatment did not ameliorate this loss (Figs. 4F, G). Interestingly, nondiabetic rats treated with minocycline showed IENF deficits compared to vehicle-treated nondiabetics (Figs. 4C, D, G; p < 0.05). Taken together these data suggest that daily minocycline does not afford significant protection against peripheral nerve dysfunction observed in experimental DPN and may negatively impact on cutaneous innervation.

### Minocycline reduces neurite outgrowth from sensory neurons

**Fig. 5:**
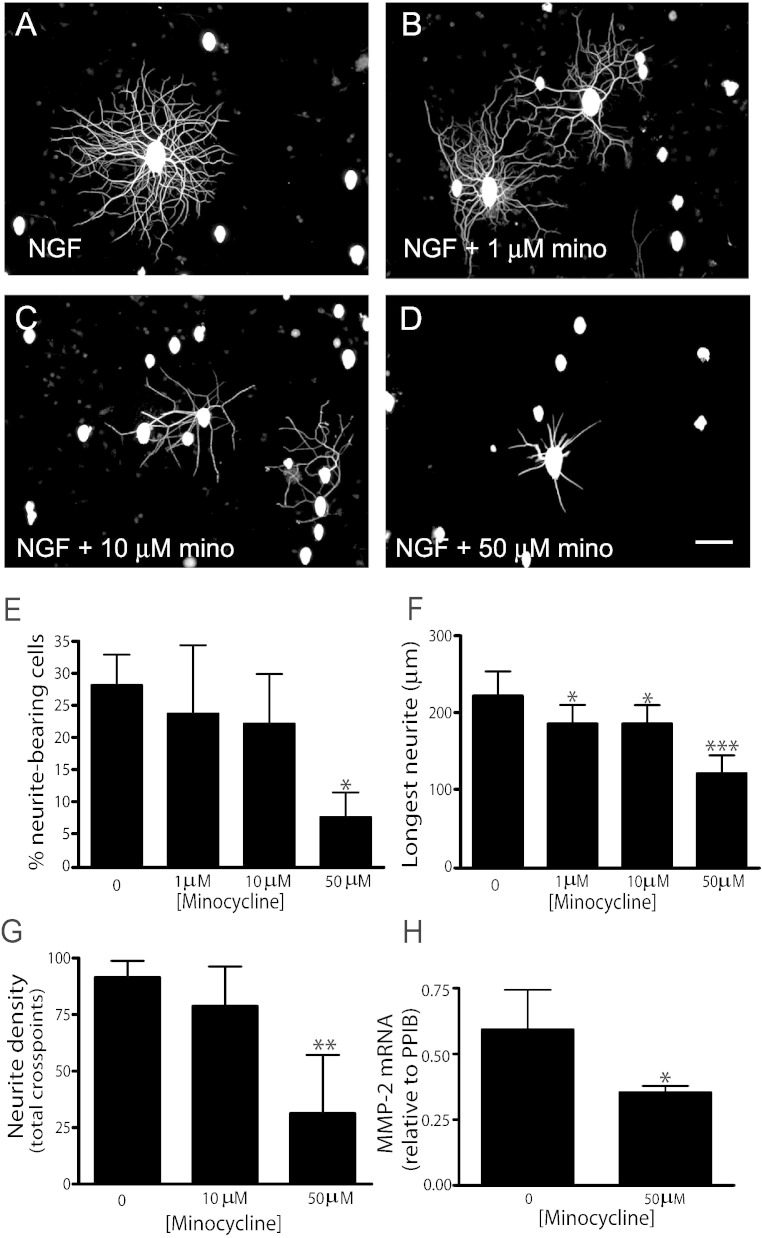
Minocycline inhibits NGF-induced neurite outgrowth. Dissociated adult rat sensory neurons extended neurites when stimulated with NGF (A; 10 ng/ml; 18 h). The addition of minocycline (B-D; 1–50 μM) led to a significant reduction in neurite initiation (E), extension (F) and density (G) (*n* = 4; * < 0.05; **p < 0.01; ***p < 0.005; one-way ANOVA followed by Bonferroni's Multiple Comparison Tests). (H) MMP-2 mRNA was significantly reduced in sensory neurons treated with NGF and 50 μM minocycline, compared to NGF alone (*p < 0.05, *t*-test). Data represent mean + s.d from at least 4 independent cultures. Scale bar = 100 μm.

The potential deleterious effects of minocycline on sensory neuron growth were investigated by the direct addition of increasing doses of minocycline to NGF-stimulated sensory neurons. Minocycline caused a reduction in NGF-mediated (Figs. 5A–D) neurite initiation (Fig. 5E. p < 0.05), elongation (Fig. 5F) and density (Fig. 5G). Neurite extension was attenuated with as little as 1 μM of minocycline (Fig. 5F, NGF alone: 222.5 ± 31 μm; 1 μM minocycline plus NGF: 186 ± 24 μm; p < 0.05). This reduction of neurite outgrowth coincided with a significant reduction in levels of MMP-2 mRNA in sensory neurons treated with minocycline (Fig. 5H, p < 0.05).

**Fig. 6:**
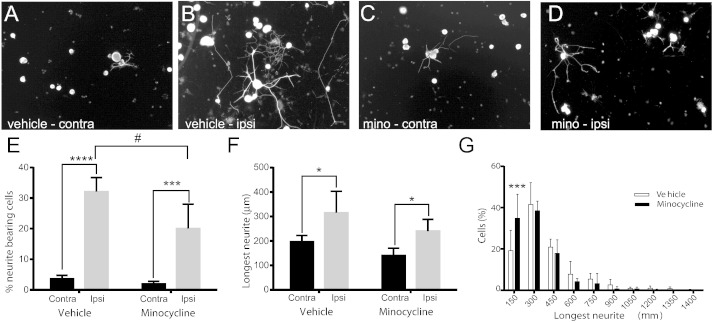
Minocycline treatment *in vivo* reduces preconditioning injury-induced neurite outgrowth *in vitro.* Control rats received a daily dose of minocycline (25 mg/kg/day, p.o; *n* = 4) or vehicle (water p.o; *n* = 4) for 3 days prior to a preconditioning sciatic nerve crush. Dosing continued for 3 days then L4/5 DRG were dissected from the nerve injured side (ipsilateral) and non-injured side (contralateral), dissociated and grown for 18 h without neurotrophic support. The neurons ipsilateral to the sciatic nerve injury (B, D) showed an increase in neurite extension compared to those from the non-injured contralateral DRG (A, C). In injured neurons, neurite initiation (E) was significantly reduced from minocycline-treated rats and neurite length distribution showed that a greater number of injured neurons from minocycline-treated rats extended significantly shorter neurites (G) compared to vehicle-treated (E–G). Scale bar = 100 μm. All data are expressed as mean ± S.D. Statistical analysis was conducted using a two-way ANOVA with Bonferroni's posthoc test. (^#^*p < 0.05, ***p < 0.001, ****p < 0.0001).

**Supplemental Fig. 1:**
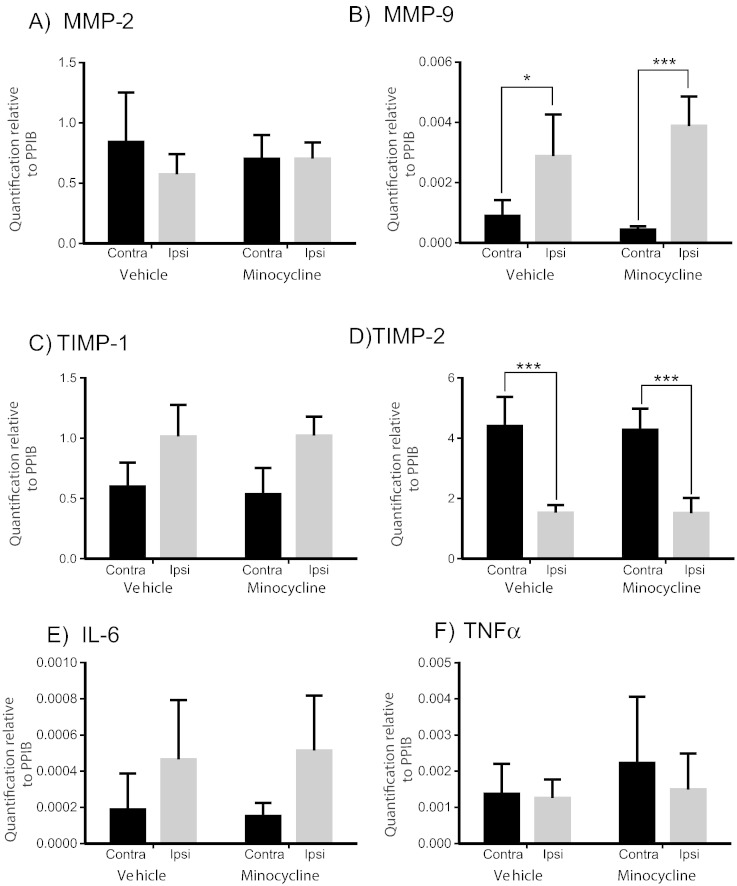
Nerve crush injury is associated with increased expression of MMP-9 and a reduction in TIMP-2 mRNA. Uninjured contralateral nerves (contra) constitutively expressed MMP-2 (A), TIMP-1 (C), TIMP-2 (D) mRNA with little detectable mRNA for MMP-9 (B) or the inflammatory cytokines IL-6 and TNF α (E, F). Three days following a nerve crush injury there was no change in MMP-2 (A), however there was a significant 3 fold decrease in ipsilateral TIMP-2 (D) and a significant increase in MMP-9 (B) in injured nerves. There was a trend towards increased IL-6, but not in TNFα at the timepoint studied (E, F). Whilst (Keilhoff et al., 2007) found daily treatment with a higher dose of minocycline (50 mg/kg) altered levels of MMP-2 mRNA 7 days following sciatic nerve injury, we found that treatment with minocycline (25 mg/kg, po) did not significantly alter these target genes 3 days following a nerve crush injury. This difference may be related to the dose of minocycline or the timepoint studied. However, our dosing over a longer time period showed a decrease in MMP-2 mRNA and protein (Fig. 7). Data represent mean + s.d (*p < 0.05; ***p < 0.001, two-way ANOVA followed by Bonferroni's Multiple Comparison Tests).

In addition, we examined whether minocycline (25 mg/kg, p.o) could affect the regenerative capacity of sensory neurons following a nerve crush injury. Injury responses, (MMP-2/-9, TIMP-1/-2, tumour necrosis factor α (TNFα) and interleukin (IL)-6)) in the sciatic nerve were measured using quantitative RT-PCR (Supp Fig. 1). We showed that vehicle- and minocycline-treated control rats had identical injury responses in the sciatic nerve. Nevertheless, preconditioning regenerative responses of sensory neurons were attenuated in minocycline-treated controls (Fig. 6). From vehicle-treated controls, there was very little neurite outgrowth from the contralateral (non-injured) neurons (Fig. 6A) whilst ipsilateral (preinjured) neurons extended numerous elongated neurites (Fig. 6B). This preconditioning injury-enhanced neurite outgrowth was attenuated in minocycline-treated controls (Figs. 6C, D) with a significant reduction in neurite initiation (Fig. 6E, ipsilateral: minocycline-treated: 20 ± 8%; vehicle-treated: 32 ± 4.6% neurite-bearing cells, p < 0.05). Whilst the overall mean length of the longest neurite was not significantly different between groups (Fig. 6F), analysis of neurite length distribution showed that a greater number of neurons from minocycline-treated controls extended significantly shorter neurites (Fig. 6G, ipsilateral: minocycline-treated: 34.8 ± 11.7% neurons extended neurites < 150 μm compared with vehicle-treated: 19.2 ± 9.9%, p < 0.001). These data highlight a negative impact of minocycline on sensory neurite outgrowth.

### Minocycline inhibits MMP-2 activity and mRNA expression in sciatic nerve

Since attenuation of neurite outgrowth by minocycline *in vitro* was associated with downregulation of MMP-2 mRNA, we examined the activity and expression of MMP-2 and TIMP-1/2 in sciatic nerve from diabetic and nondiabetic rats treated with either vehicle or minocycline.

**Fig. 7:**
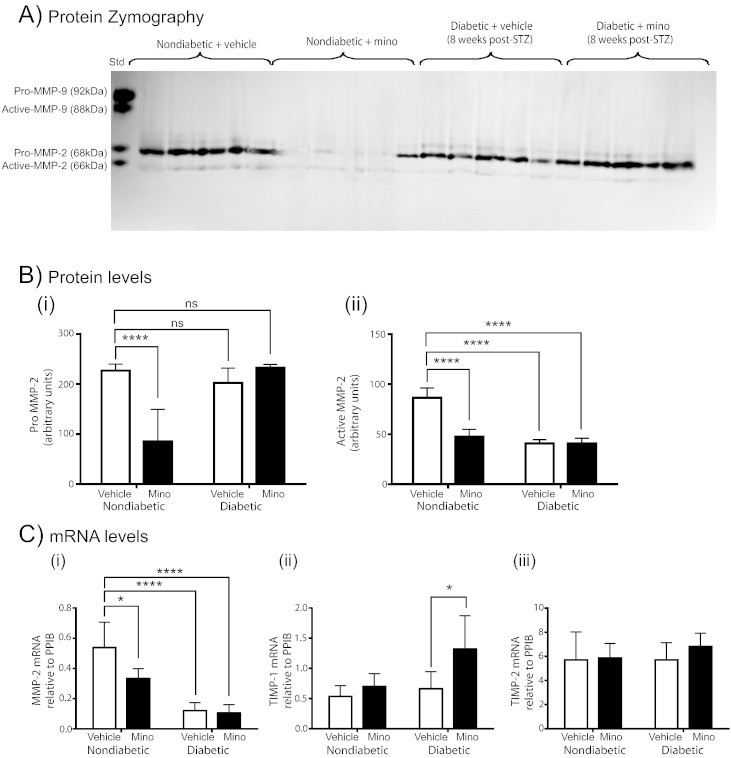
Levels of active MMP-2 decrease in the sciatic nerve with diabetes and minocycline-treatment. Age-matched nondiabetic and STZ-diabetic rats were treated with minocycline (25 mg/kg p.o.) daily from 3 days post-STZ for 8 weeks. Proteolytic activity of MMP-2 and MMP-9 were assayed using gelatin zymography, each lane contained sciatic nerve protein from an individual animal (*n* = 5 per group; std: MMP-9 and MMP-2 protein standard to act as positive control/marker). Bands corresponding in molecular weight to the pro- and active-cleaved forms of MMP-2 were detected (A). Densitometric analysis (B i, ii) revealed a diabetes-associated decrease in active MMP-2 which was not altered by minocycline. However, minocycline treatment of nondiabetic rats reduced both pro and active MMP-2 (A, B i,ii). Quantitative RT-PCR analysis (C i–iii) revealed reduced MMP-2 mRNA with minocycline treatment and also with diabetes. There was no change in levels of TIMP-2 (C iii), but levels of TIMP-1 mRNA (C ii) were increased in nerves from minocycline-treated diabetic rats. Data represent mean + s.d (*p < 0.05; ****p < 0.0001, two-way ANOVA followed by Bonferroni's Multiple Comparison Tests).

By zymography we confirmed active MMP-2 levels were again reduced in sciatic nerves from vehicle-treated diabetic compared to vehicle-treated nondiabetic rats (Figs. 7A, B (ii), p < 0.0001). Minocycline treatment of diabetic rats did not further affect the reduced levels of sciatic nerve MMP-2 (Fig. 7B (ii)). However, minocycline treatment of nondiabetics reduced levels of both the pro and active MMP-2 compared to vehicle-treated nondiabetics (Figs. 7A, B (i, ii), p < 0.0001). By RT-PCR we show the expression of MMP-2 was significantly reduced in nerves from diabetic rats (Fig. 7C (i), p < 0.0001) and in minocycline-treated nondiabetics compared with vehicle-treated nondiabetics (Fig. 7C (i), p < 0.05). Minocycline treatment of diabetic rats was associated with a significant increase in the expression of TIMP-1 (Fig. 7C (ii) p < 0.05) with no change in TIMP-2 mRNA (Fig. 7C (iii)).

These data suggest that in diabetes MMP-2 is downregulated at the level of transcription and by reduced cleavage of the pro-form to the active form and/or interactions of the active form with TIMPs. In nondiabetic rats minocycline modulates MMP-2 by direct enzymatic inhibition and transcriptional alterations in both MMP-2 and TIMP-1 mRNA.

## Discussion

In summary, we confirmed that MMP-2 but not MMP-9 was constitutively expressed in both pro and active forms in the adult rat sciatic nerve (Ferguson and Muir, 2000, Shubayev and Myers, 2000). The expression and activity of MMP-2 (mRNA and protein) were downregulated by either treatment with minocycline (an inhibitor of MMPs) and/or induction of diabetes. The functional consequences of this downregulation were a small but statistically significant reduction in IENF density in minocycline-treated nondiabetic rats, and in diabetes the sciatic nerve was less supportive of sensory neuron growth. The inhibitory effects of minocycline on regeneration were further demonstrated by impaired preconditioning-injury-induced neurite outgrowth, and its direct addition to sensory neurons impaired NGF-mediated neurite outgrowth. In contrast, pretreatment of sciatic nerve substrata from diabetic rats with active MMP-2 or the direct addition of active MMP-2 to sensory neuron cultures promoted neurite elongation, potentially by increasing ECM turnover and/or increasing the local release of neurotrophins.

We examined the expression and activity of MMPs in the sciatic nerve over a 12-week timecourse of diabetes and found downregulation of both MMP-2 mRNA and levels of active MMP-2 in sciatic nerve. In addition we found no constitutive or diabetes-associated expression of MMP-9, but confirmed MMP-9 upregulation following nerve injury (Platt et al., 2003). Our results differ from those reported by (Bhatt and Veeranjaneyulu, 2010) who showed increased levels of both MMP-2 and MMP-9 in sciatic nerve 6 weeks post STZ injection. Whilst we did not study this timepoint, we propose that an early transient increase in MMPs may reflect initial attempts of nerve regeneration and the inability to maintain increased activity of MMP-2/-9 from 8 weeks onwards may explain the delays in Wallerian degeneration and failure of nerve regeneration in diabetes (Kennedy and Zochodne, 2005). In diabetes the increased formation of advanced glycation residues (AGEs) and altered properties of glycated ECM molecules produce similar downregulation of MMP expression in other tissues; including MMP-7 in mesangial cells in diabetic nephropathy (McLennan et al., 2007) and downregulation of MMP-1/-2 in dermal fibroblasts (Rittie et al., 1999). We have previously shown that AGE residues are increased in ECM of sciatic nerve from STZ-diabetic rats (Duran-Jimenez et al., 2009) and suggest that in addition to compromising protein function through crosslinking they may also negatively regulate MMP-2 expression and activity.

We have shown that sciatic nerve substrata prepared from diabetic rats were less supportive of sensory neuron growth. Pretreatment of nerves with active MMP-2 rescued neurite elongation to levels observed on nondiabetic rat nerve substrata, suggesting that reduced levels of MMP-2 together with increased levels of AGEs may contribute to the accumulation of collagen and ECM molecules in peripheral nerve (Bradley et al., 2000, Giannini and Dyck, 1995, Hill and Williams, 2004, King et al., 1989). Furthermore the addition of active MMP-2 enhanced neurite outgrowth from dissociated adult sensory neurones, potentially through increased release of NGF. In diabetic retinopathy altered expression and activity of MMP-7 has been shown to impair the homeostatic balance between the pro-form and cleaved active form of NGF and contribute to neurovascular dysfunction (Ali et al., 2011). Since NGF deficits have been described in diabetic neuropathy (Anand et al., 1996, Hellweg and Hartung, 1990) we propose that reduced MMP-2 levels may directly alter the availability of active neurotrophins in diabetes. Collectively our data illustrates that reduced levels of active MMP-2 (and absence of MMP-9) may contribute to a number of the phenotypic dysfunctions observed in DPN.

Attempts to treat DPN can be divided into those directed at modification of the underlying disease process and those directed at symptom suppression. The analgesic potential of minocycline has been described in experimental models of chemotherapy-induced polyneuropathy (CIPN; (Boyette-Davis and Dougherty, 2011, Boyette-Davis et al., 2011), DPN (Bhatt and Veeranjaneyulu, 2010, Morgado et al., 2011, Pabreja et al., 2011, Zychowska et al., 2013) and nerve injury (Mika et al., 2007). In these studies minocycline was administered at a wide range of doses (100 μg–100 mg/kg) by a number of routes including intraperitoneal, intrathecal and gavage. Minocycline inhibits several enzymes acting within the apoptosis- and inflammatory-pathways (MMP-2/-9, iNOS and COX), inhibits microglial cell activation, alters the expression of inflammatory cytokines, scavenges reactive oxygen species and alters potassium-chloride cotransporter-2 in the spinal cord.

We administered minocycline orally at a dose of 25 mg/kg a route and dose regimen which has previously been shown to cross the blood brain barrier (Colovic and Caccia, 2003) and protect against IENF loss in CIPN (Boyette-Davis and Dougherty, 2011, Boyette-Davis et al., 2011). Following 8 weeks of daily minocycline-treatment we observed reduced levels of both pro and active MMP-2 protein and mRNA in sciatic nerve from nondiabetic-treated rats. In diabetic rats the already reduced levels of active MMP-2 in sciatic nerve were not further impacted on by minocycline treatment. Interestingly nerves from minocycline-treated diabetic rats showed upregulation of TIMP-1 mRNA, possibly indicating an altered pharmacokinetic profile and altered MMP/TIMP regulation between nondiabetic and diabetic rats. Administration of minocycline at a higher dose (50 mg/kg, p.o) however has been shown to reduce MMP-2/-9 levels in sciatic nerve of diabetic rats (6 weeks post STZ (Bhatt and Veeranjaneyulu, 2010)) and following peripheral nerve injury (Keilhoff et al., 2007). In our study minocycline did not afford significant protection against large (NCV) and small fibre (IENF) deficits in DPN. NCV measurements were made one day following the last dose of minocycline, therefore it is possible that any transient protective effects of minocycline on NCV were not detected. Surprisingly, treatment of nondiabetic rats was associated with a small, but statistically significant, reduction in IENF density. The negative effects of minocycline on sensory nerve regeneration were further demonstrated by the ability of *in vivo* treatment of control rats to attenuate the preconditioning injury response of dissociated DRG following a crush injury, and *in vitro* treatment to directly inhibit NGF-mediated neurite outgrowth. This reduction in growth was associated with reduced expression of MMP-2 mRNA.

Our studies add to the increasing evidence demonstrating the variable and even detrimental effects exerted by minocycline on neuronal and glial cells in a number of neurological diseases. For example, minocycline did not improve functional outcome or regeneration following a rat spinal contusion injury (Diguet et al., 2004, Lee et al., 2003) and worsened patient outcome in a clinical trial for amyotrophic lateral sclerosis (Gordon et al., 2007). In organotypic rat spinal cord cultures, high doses of minocycline were deleterious to motor neuron survival and impaired activation, survival and migration of microglia and astrocytes (Pinkernelle et al., 2013). Minocycline treatment, in a sciatic nerve transection/immediate graft repair injury model, showed impaired axonal regeneration, associated with reduced macrophage recruitment and activation, retarded Wallerian degeneration and reduced levels of MMP-2/9 and iNOS in the nerve graft (Keilhoff et al., 2007). Importantly a few clinical case reports have described neuropathy as an adverse event of minocycline treatment (ulnar neuropathy, carpel tunnel syndrome, vasculitic neuropathy, axonal neuropathy) (Lawson et al., 2001, Thaisetthawatkul et al., 2011, Morgado et al., 2011, Palamaras et al., 2008).

We hypothesise that the beneficial *versus* detrimental effects of minocycline treatment depend on the balance of targets that it interacts with. In the absence of pathology, minocycline inhibits the constitutive activity of MMPs in sciatic nerve which is essential for the maintenance of nerve health and regeneration. In disease, alterations in levels of MMPs, inflammation, oxidative stress and apoptosis means there is potentially a wider range of targets for minocycline to interact with. The exact contributions of these to the disease pathology and also timing of treatment will determine therapy outcomes with minocycline. Further experiments using minocycline in DPN, especially at the higher doses (40–80 mg/kg) used to alleviate hyperalgesia or allodynia (Bhatt and Veeranjaneyulu, 2010, Morgado et al., 2011, Pabreja et al., 2011), are required to characterise any negative impact of minocycline on IENF density.

## Conclusion

We show that levels of active MMP-2 are downregulated in peripheral nerve during diabetes and with treatment with minocycline. We suggest that loss of MMP-2 is associated with ECM abnormalities and reduced neurotrophin availability, which contributes to nerve degeneration and IENF loss. Thus we propose that strategies to activate MMP-2 may improve the regenerative capacity of peripheral nerve in diabetes. Whilst our studies do not exclude the possible beneficial effects of minocycline in painful DPN, the present study does question its long-term use in a syndrome where there is ongoing nerve pathology and nerve regeneration is necessary to preserve or restore nerve function.

The following are the supplementary data related to this article
